# *CSF1* Is a Novel p53 Target Gene Whose Protein Product Functions in a Feed-Forward Manner to Suppress Apoptosis and Enhance p53-Mediated Growth Arrest

**DOI:** 10.1371/journal.pone.0074297

**Published:** 2013-09-03

**Authors:** Gregory Azzam, Xuting Wang, Douglas Bell, Maureen E. Murphy

**Affiliations:** 1 Program in Molecular and Cellular Oncogenesis, The Wistar Institute, Philadelphia, Pennsylvania, United States of America; 2 Laboratory of Molecular Genetics, Intramural Research Program, National Institute of Environmental Health Sciences-National Institutes of Health, Research Triangle Park, North Carolina, United States of America; Rush University Medical Center, United States of America

## Abstract

The p53 tumor suppressor gene has a common polymorphism at codon 72 that alters its function. We previously reported that the proline 72 polymorphic variant of p53 (P72) demonstrates increased ability to transactivate a subset of genes, relative to arginine 72 (R72); one of these genes is macrophage colony stimulating factor (*CSF1*). At present, the mechanism(s) underlying the increased transcriptional activity of P72 toward genes like *CSF1* have not been completely elucidated. Additionally, the consequences of increased transcription of genes like CSF1 by the P72 variant to the downstream p53 pathway are unknown. In this report, we address these issues. We show that the *CSF1* gene contains a conserved binding site for p53, and interestingly that the P72 variant shows increased ability to bind to this site. Moreover, we show that increased CSF1/CSF1R signaling in P72 cells feeds back on the p53 pathway to enhance p53 phosphorylation, levels, and transactivation of target genes, particularly the cyclin-dependent kinase inhibitor p21 (*CDKN1A*). This leads to an increase in p53-mediated growth arrest, along with a concomitant decrease in apoptosis. Notably, the CSF1/CSF1R signaling axis is overexpressed in several epithelial cancers, and there is clinical evidence that this pathway plays a role in radio-resistance of some cancers. We show that cells expressing CSF1 and CSF1R are indeed radio-resistant, and further, that this effect requires p53. These combined data are the first to implicate the CSF1/CSF1R pathway in the decision between p53-mediated growth arrest and apoptosis. They are also the first to highlight a cytokine as influential in cell fate determined by p53 in epithelial cells. Finally, these data may explain the association of the P72 variant and the CSF1/CSF1R pathway with increased senescence and radio-resistance in some epithelial tumor types.

## Introduction

The p53 tumor suppressor gene is a key player in the cellular response to stress. p53 responds to a variety of detrimental environmental conditions, including DNA damage, oxidative stress, hypoxia, nutrient deprivation, and oncogene activation. The stabilized protein responds to these stresses by inducing either growth arrest (transient or permanent) or apoptosis. Currently, the decision between growth arrest and apoptosis mediated by p53 is not completely understood. In many instances this decision is cell-type specific [1], [2], [3]. Additionally, the decision can be influenced by the level of p53 induced [4], the ratio of expression of BCL2 family members [5], or by post-translational modifications on p53 [6], [7]; for review see [8]. Finally, in a few hematopoietic cell lines, the presence of survival cytokines inhibits p53-mediated apoptosis, and this allows for p53-mediated growth arrest to occur [9], [10]. Cytokines that influence the growth arrest versus cell death decision by p53 in epithelial cell types and cancers have not been identified.

A common polymorphism in the p53 gene encodes either proline or arginine at amino acid 72 (P72 or R72, respectively). Studies in cultured cells containing endogenous or inducible versions of these variants indicate that the R72 allele possesses enhanced apoptotic function, associated with increased mitochondrial localization [11] and possibly also enhanced ability to transactivate a subset pro-apoptotic p53 target genes [12], [13]. Conversely, the P72 variant demonstrates increased ability to transactivate the cyclin-dependent kinase inhibitor p21/waf1 (*CDKN1A*), along with increased ability to induce growth arrest and senescence [14], [15], [16]. Interestingly, the P72 variant is associated with increased longevity in humans, and shows increased incidence in Centenarians; this may be related to the increased ability of this variant to induce growth arrest over apoptosis [16], [17]. Recently, our group and another group independently generated mouse models for the human P72 and R72 variants. Notably, in both models, the behavior of these variants completely recapitulated the findings regarding these polymorphic variants in human cells. Specifically, the P72 variant showed increased ability to transactivate p21 (CDKN1A) and induce senescence, while the R72 variant showed increased ability to induce apoptosis [15], [18], though the latter effect only occurred in certain tissues [19].

Despite the fact that this polymorphism clearly alters p53′s ability to induce growth arrest and apoptosis, an impact of the codon 72 polymorphism on human cancer incidence has not been obvious. In general, meta analyses have failed to find compelling evidence for an association of this polymorphism with overall cancer risk [20], [21]. Interestingly, however, there has been clear evidence for association of the P72 polymorphism with diabetes [22], [23] and colitis [24], [25], [26], two diseases that can confer increased cancer risk. These data suggest that, whereas overall cancer risk may be unaffected, more specific cell-type or tumor-specific behaviors may be affected by the codon 72 polymorphism. In this study, we show that radio-sensitivity is influenced by the codon 72 polymorphism, and that this is related to the differential ability of these variants to signal to, and respond to, the CSF1/CSF1R pathway. These data suggest that an effect of this polymorphism on the radiation response of tumors with wt p53 should be analyzed, for example in breast cancers that overexpress the CSF1/CSFR1 pathway.

## Results

### The Proline 72 (P72) Variant of p53 Shows Increased Transactivation of CSF1, Compared to Arginine 72 (R72)

**Figure 1:**
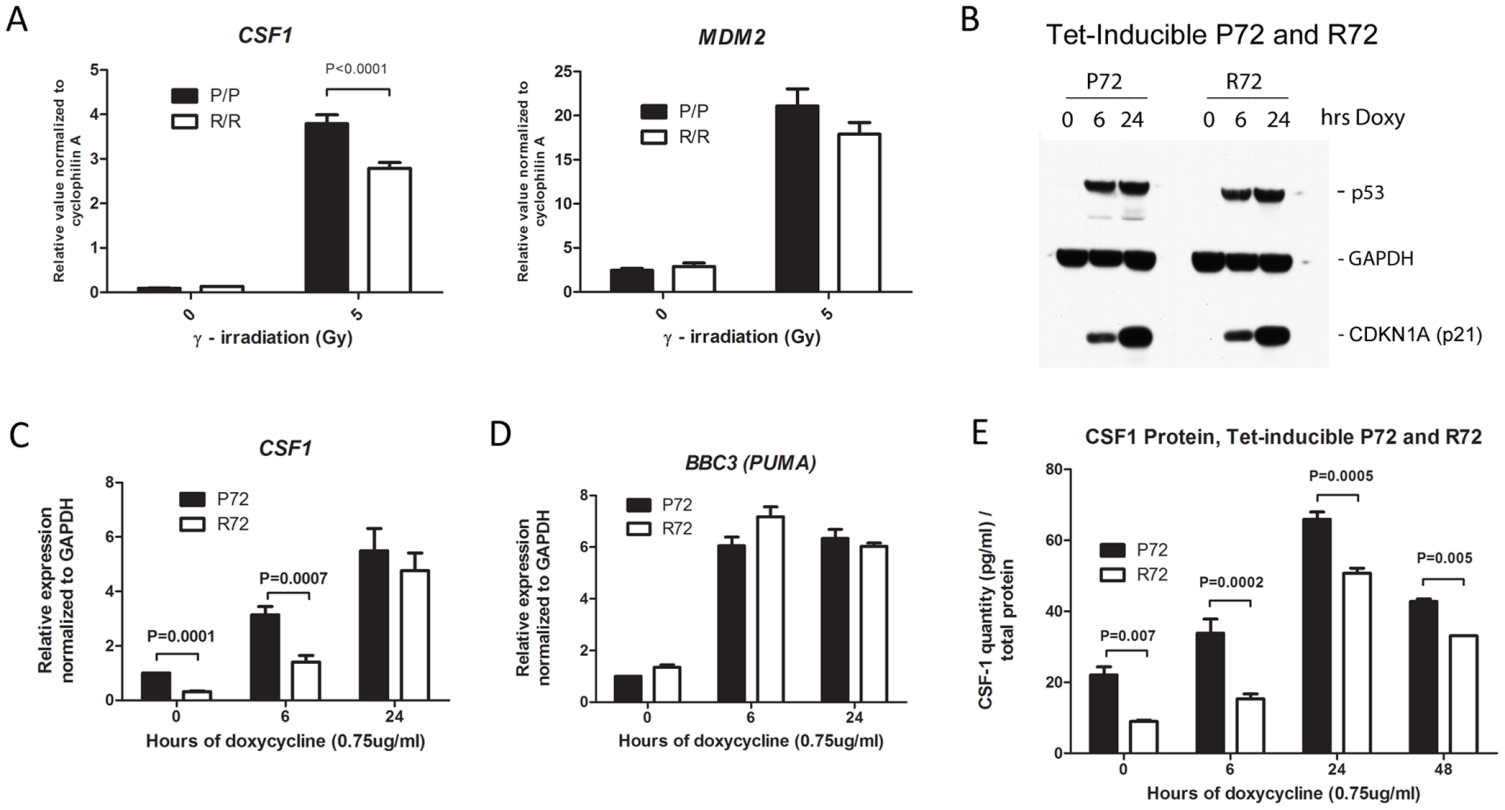
Increased CSF1 expression induced in cells expressing the P72 variant of p53. A. Quantitative reverse transcription/polymerase chain reaction (QRT-PCR) analysis of RNA isolated from the thymuses of P72 and R72 Hupki mice, analyzed for the level of CSF1 and MDM2 following treatment of mice with 5 Gy gamma radiation after 4 hours. Values shown are the average of independent experiments in which a total of three homozygous P72 (P/P) and R72 (R/R) mice (n = 3 each genotype for treated, 1 each genotype for untreated) were analyzed following radiation; levels are normalized to cyclophilin A. Gy = Gray. Error bars mark standard error. B. Western analysis of lysates from H1299 cells containing tetracycline (tet) inducible p53 encoding either the P72 or the R72 variant for the levels of p53, GAPDH and p21 (CDKN1A). Cells were treated with 0.75 ug/ml doxycycline (doxy) for the indicated time points (hrs). C. QRT-PCR of RNA isolated from tet-inducible p53 P72 or R72 cells treated with doxycycline (doxy) for the indicated time points and analyzed for the level of CSF1 mRNA; values shown are the average of 4 independent experiments, normalized to GAPDH. The error bars mark standard deviation. D. QRT-PCR of RNA isolated from tet-inducible p53 P72 or R72 cells treated with doxycycline (doxy) for the indicated time points and analyzed for the level of BBC3 (PUMA); values shown are the average of four independent experiments, normalized to GAPDH. The error bars mark standard deviation. E. Enzyme-linked immunosorbent assay (ELISA) analysis of CSF1 protein level in lysates isolated from H1299 cells with tetracycline-inducible p53 (P72 or R72) untreated or treated with doxycycline (0.75 ug/mL) for the indicated time points. The data depicted are the averaged results from three independent experiments; error bars mark standard error.

We previously created humanized p53 knock-in mice for both codon 72 variants of p53, proline 72 (P72) and arginine 72 (R72), and showed that these variants functionally recapitulate human phenotypes in mice [15], [19]. In that same study, we performed a micro-array analysis to identify genes that were differentially regulated by the P72 and R72 variants of p53 in the irradiated mouse thymus. This analysis revealed that over 95% of p53 target genes displayed identical transactivation by the P72 and R72 polymorphic variants. However, we identified a subset of approximately a dozen genes that showed increased transactivation by the P72 variant of p53 [15]. One of these genes, macrophage colony stimulating factor 1, or *CSF1*, was selected here for further analysis. We first sought to confirm the micro-array data by analyzing CSF1 gene expression in age-matched littermate P72 and R72 Hupki mice generated from P/R heterozygous crosses. Three sibling mice homozygous for each genotype (PP or RR) were subjected to 5 Gy gamma radiation, and RNA was isolated from purified thymocytes 4 hours after radiation. Quantitative reverse-transcription/polymerase chain reaction (QRT-PCR) analyses indicated that, consistent with our previous findings, P72 mouse thymocytes expressed significantly higher levels of CSF1 mRNA relative to R72 after gamma irradiation (3.8+/−0.2 vs. 2.8+/−0.1, p = 0.0001). In contrast, transactivation of another p53 target gene, MDM2, showed no difference between the variants (**Fig. 1A**), nor did transactivation of several other p53 target genes (data not shown).

We next sought to determine if the increased ability of the P72 variant of p53 to transactivate CSF1 was also evident in human cells. We were unable to detect CSF1 expression in normal human fibroblasts or in melanoma cell lines that we have genotyped for P72 or R72 (G. Azzam, unpublished data). Therefore, we sought to address this question by analyzing CSF1 transactivation in a model system composed of H1299 lung adenocarcinoma cells containing tetracycline-inducible versions of either p53 variant, P72 or R72. These lines have been selected for equal levels of p53 induction, and treatment with doxycycline for 6 or 24 hours led to an accumulation of nearly identical levels of p53 protein (**Fig. 1B**). QRT-PCR analysis of the RNA levels of CSF1, as well as the p53 target gene BBC3 (PUMA), revealed that there was significantly greater transactivation of CSF1 by the P72 variant, compared to R72, 6 hours after p53 induction with doxycycline (p = 0.0007, **Fig. 1C**), though by 24 hours this difference was largely diminished. In contrast, the transactivation of BBC3 (PUMA) by both p53 variants was indistinguishable (**Fig. 1D**). We noted a slight difference in CSF-1 level in uninduced P72 and R72 cells; this is likely due to slight leakiness of this inducible system, as we were able to detect a low level of p53 protein even in the absence of doxycycline (G. Azzam, our unpublished data). To analyze the CSF1 protein level in these cell lines, we performed ELISA assays on lysates generated from doxycycline-inducible P72 and R72 cells. These analyses revealed a consistent increase in CSF1 protein in lysates from P72 cells, compared to R72, at all time points analyzed (**Fig. 1E**). The combined data indicate that in murine thymocytes expressing P72 and R72 variants, as well as in human inducible cell lines expressing both variants, the P72 variant of p53 shows consistently enhanced ability to transactivate CSF1, but not other p53 target genes.

### CSF1 is a Direct P53 Target Gene

**Figure 2:**
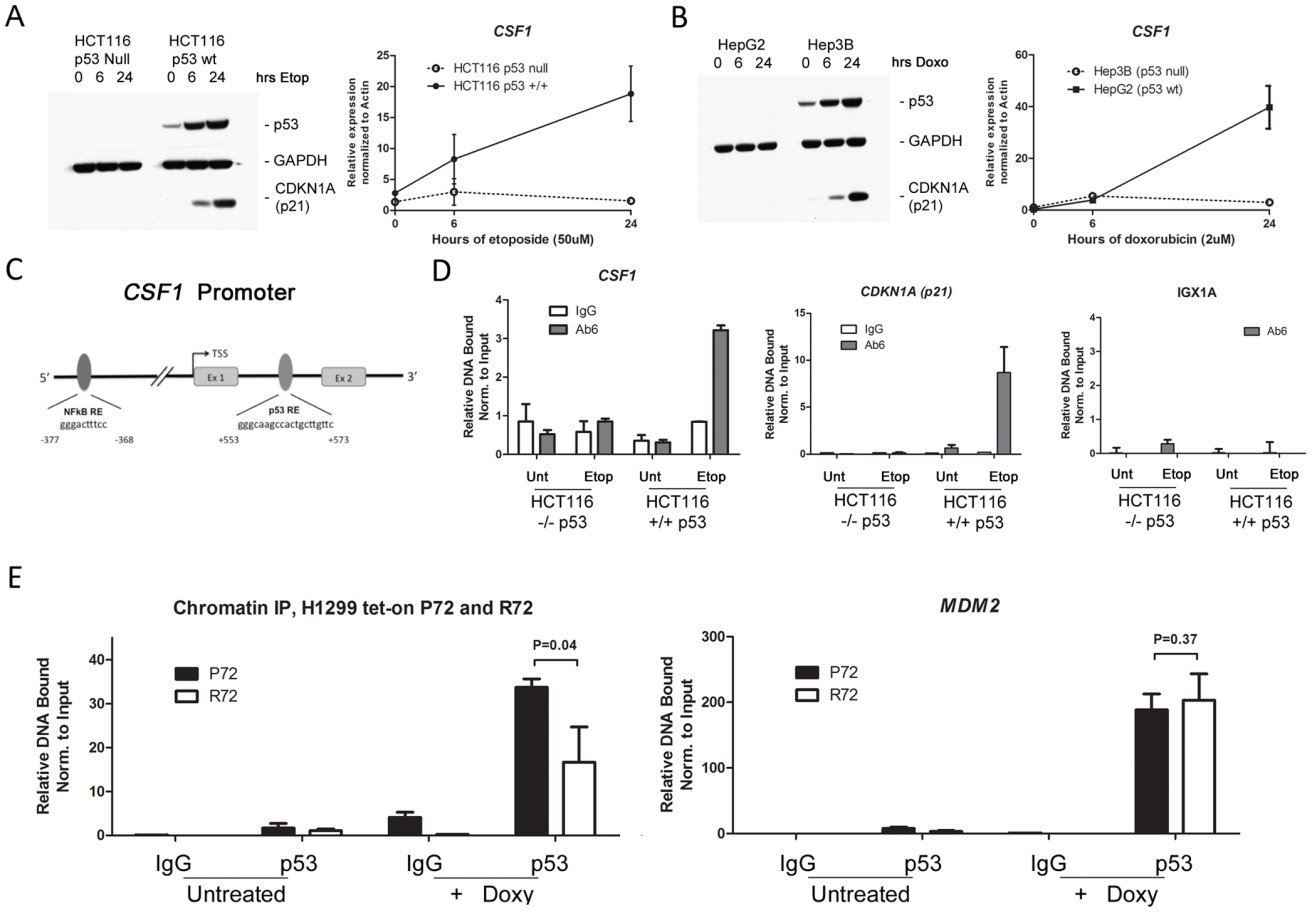
CSF1 is a novel p53 target gene. A. Left panel: Western blot analysis of the level of p53, GAPDH and p21 (CDKN1A) in lysates isolated from Hct116 cells (p53−/− or wild-type p53, +/+), untreated or treated with Etoposide (Etop, 50 uM) for the indicated time points. Right panel: QRT-PCR analysis of the level of CSF1 RNA in the samples depicted in the left panel; values shown are the average of 3 independent experiments normalized to control. Error bars mark standard deviation. B. Left panel: Western analysis of the level of p53, GAPDH and p21 in lysates isolated from Hep3B (p53−/−) or HepG2 (wt p53) untreated or treated with 2 uM Doxorubicin (Doxo) for the indicated time points. Right panel: QRT-PCR of RNA isolated from the samples in the left panel, untreated or treated with 2 uM Doxorubicin (Doxo) for the indicated time points (hours, hrs), analyzed for CSF1. Values shown are the average of 3 independent experiments normalized to control. Error bars mark standard deviation. C. Schematic of the Human *CSF1* promoter, where the transcription start site (TSS) is denoted nucleotide +1, and the presence of canonical NF-kB and p53 response elements (RE) are denoted. Ex 1: exon 1. Ex 2: exon 2. D. Quantitative PCR (Q-PCR) analysis of Chromatin Immunoprecipitation (ChIP) eluates isolated from Hct 116 p53+/+ or −/− cells, untreated or treated with Etoposide (Etop, 50 uM) for 18 hours. ChIP was performed using antibody to p53 (Ab6, Calbiochem) or the equivalent amount of normal mouse Immunoglobulin G (IgG). Primer sets used for Q-PCR flank the predicted p53 response element in the CSF1 promoter depicted in (C), the positive control p53 response element in the p21 promoter (CDKN1A), and negative control primers (IGX1A, Qiagen), as indicated in the Materials and Methods. Values shown are the average of 2 independent experiments each repeated in duplicate, and are presented as the percent DNA bound normalized to input. E. Quantitative PCR (Q-PCR) analysis of Chromatin Immunoprecipitation (ChIP) eluates isolated from tetracycline-inducible H1299 cells, P72 or R72, untreated or treated with doxycycline (0.75 ug/ml) for 18 hours. ChIP was performed using antibody to p53 (fl393, Santa Cruz Biotechnology) or an equivalent amount of normal rabbit Immunoglobulin G (IgG). Primers used for Q-PCR analysis flank the predicted p53 response element on the CSF1 promoter, or the known p53 response element in the MDM2 promoter. Values shown are the average of 2 independent experiments each repeated in duplicate, and are presented as the relative DNA bound normalized to input.

We next sought to determine whether CSF1 is a direct target for transcriptional activation by p53. We first analyzed two pairs of cell lines that differ in p53 status for the level of CSF1 induced after exposure to DNA damaging agents. In the colorectal carcinoma Hct116 p53+/+ and −/− pair, etoposide treatment led to efficient stabilization of p53, induction of p21 (CDKN1A), and induction of CSF1 mRNA, all in a p53-dependent manner (**Fig. 2A**). Similarly, in Hep3B (p53 null) and HepG2 (wt p53) liver cell lines, CSF1 was induced by the DNA damaging agent doxorubicin in a p53-dependent manner (**Fig. 2B**). Analysis of the promoter and intronic regions of CSF1 using a position weight matrix (PWM) method [27] revealed the presence of two overlapping consensus p53 binding sites in intron 1 of this gene (**Fig. 2C** and **Fig. S1**). Consistent with this being an authentic p53 binding site, ChIP-seq data from lymphoblastoid cell lines treated with doxorubicin demonstrated a binding peak over this region for p53 (**Fig. S1**). Similarly, published p53 ChIP-seq data from U2OS cells treated with etoposide and Actinomycin D showed a weak but detectable binding peak in this region [28]. We performed chromatin immunoprecipitation in the Hct116 pair treated with etoposide. These experiments revealed that p53 antisera could consistently immunoprecipitate DNA surrounding this p53 binding site in Hct116 cells, but not in p53−/− cells (**Fig. 2D**). In contrast, oligonucleotides for IGX1A, which maps to a promoter desert, were negative for p53 binding, as were oligonucleotides for a region far downstream of the p53 binding site in CSF1 (**Fig. 2D** and data not shown). To begin to assess the mechanism whereby the P72 variant shows enhanced transactivation of CSF1 relative to R72, we next performed chromatin immunoprecipitation in tetracycline-inducible P72 and R72 cells using p53 antisera, and noted an approximately 2-fold increase in the ability of the P72 variant to ChIP the CSF1 consensus p53 binding site, relative to R72; in contrast, these two variants bound identically to the p53 binding site in the MDM2 promoter (**Fig. 2E**). Notably, increased binding of P72 was consistent using two different p53 antibodies (**Fig. S2**). The combined data support the conclusion that CSF1 is a direct p53 target gene, and that at least part of the mechanism for enhanced transactivation by P72 may involve increased DNA binding of this variant to this element in the CSF1 promoter.

### Transactivation of CSF1 by p53 Does not Require NF-kB

**Figure 3:**
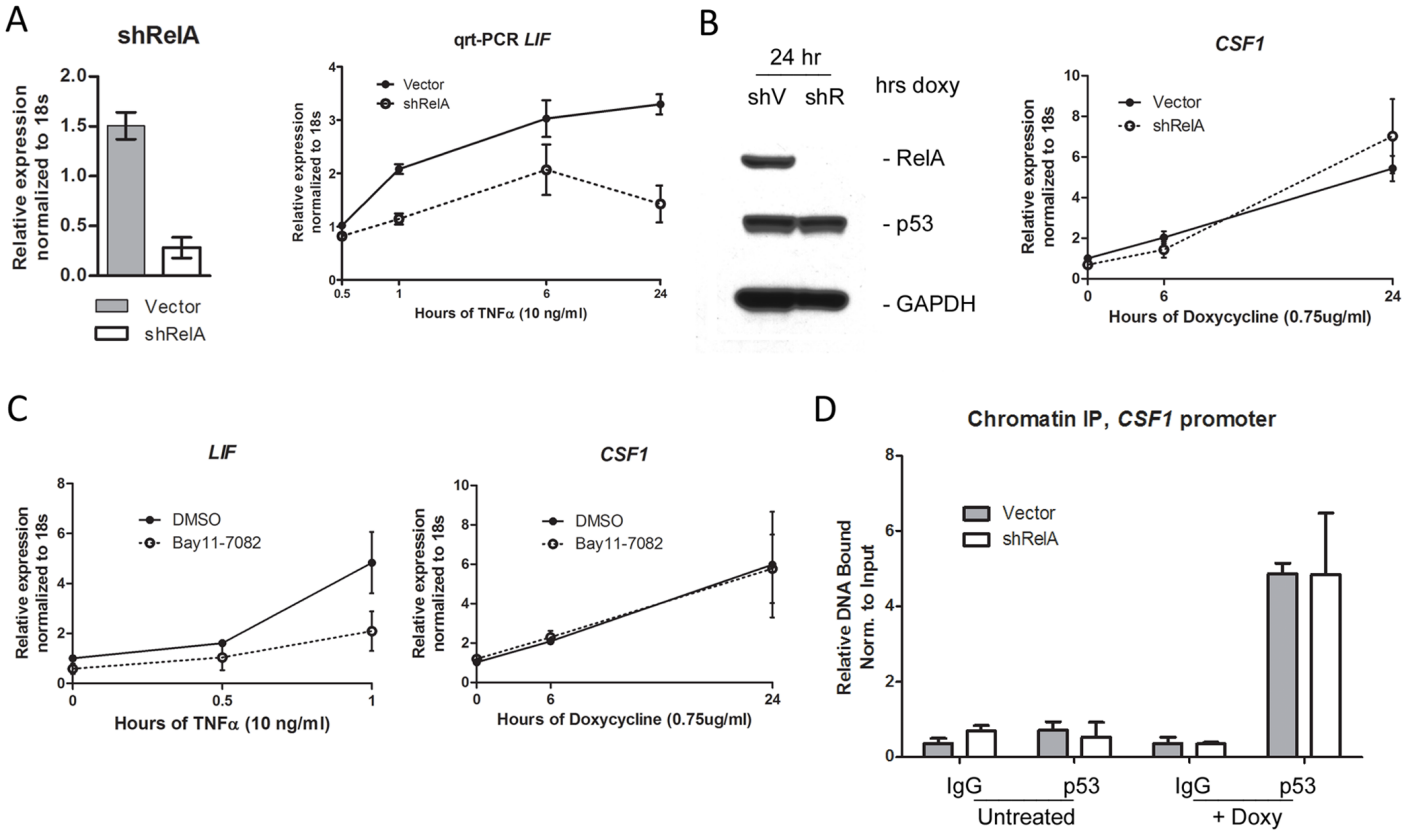
The NF-kB subunit p65RelA does not play a role in the transactivation of CSF1 by p53. A. Left panel: QRT-PCR of p65RelA RNA level in H1299 tet-inducible p53 (P72 variant) cells following infection with a short-hairpin to RelA (shRelA) or vector alone (Vector). Values shown are the average of 3 independent replicates, normalized to control. Right panel: QRT-PCR of RNA isolated from H1299 tet-inducible p53 cells (P72 variant), infected with shRelA or vector. Cells were treated with TNF-α (10 ng/mL) and analyzed for the level of LIF after the indicated time points (hours). Values shown are the average of 3 independent replicates, normalized to control. Error bars mark standard deviation. B. Left panel: Western analysis of lysates isolated from H1299 tet-inducible p53 (P72 variant) cells following infection with a short-hairpin to RelA (shRelA) or vector alone (Vector), after 24 hours of doxycycline treatment (0.75 ug/ml); lysates were analyzed for the level of p53, p65RelA and GAPDH. Right panel: QRT-PCR of RNA isolated from shRelA or Vector tet-on p53 H1299 cells, analyzed for CSF1 after no treatment (0), or treatment with doxycycline (0.75 ug/ml) for the indicated time points (hours). Values shown are the average of 4 independent replicates, normalized to control. Error bars mark standard deviation. C. QRT-PCR of RNA isolated from H1299 tet-inducible p53 cells (P72 variant), infected with shRelA or vector. Cells were untreated (0 hr) or treated with Bay11–7082 or DMSO just prior to the addition of TNF-α (10 ng/ml) for the indicated time points. RNA was analyzed for the levels of LIF and CSF1; values shown are the average of 3 independent replicates, normalized to control. Error bars mark standard deviation. D. Q-PCR of chromatin immunoprecipitation (ChIP) eluates isolated from shRelA or Vector tet-inducible p53 cells, untreated or treated with doxycycline (0.75 ug/ml) for 18 hours. Immunoprecipitations were performed using antibody to p53 (Ab6) or the equivalent amount of normal mouse Immunoglobulin G (IgG). Primers used for Q-PCR analysis flank the predicted p53 response element on the CSF1 promoter. Values shown are the average of 2 independent experiments repeated in duplicate, and are presented as percent bound normalized to input. Error bars mark standard error.

In order to assess a possible mechanism for increased P72 binding, we next sought to test the hypothesis that NF-kB might influence p53 binding to the CSF1 promoter. We previously showed that the P72 variant of p53 demonstrates increased ability to interact with the p65RelA subunit of NF-kB, compared to R72. Further, we showed that this enhanced interaction was responsible in part for the increased ability of P72 to transactivate the gene for murine caspase-11, which contains both p53 and NF-kB consensus elements in its promoter [15]. In support of this premise, we previously reported that silencing p65RelA reduced the ability of the P72 variant to transactivate caspase-11 [15]. We noted a consensus NF-kB element in the promoter of CSF1 (**Fig. 2C**), and therefore sought to test the hypothesis that a similar cooperation between P72 and NF-kB might exist for the CSF1 gene, and thus further explain the mechanism for enhanced transactivation of this gene by P72. To address this hypothesis, we generated H1299 cells containing tetracycline-inducible P72 protein that were stably infected with control lentivirus (Vector) or a lentivirus expressing a short hairpin to p65RelA (shRelA). QRT-PCR analysis indicated that the short hairpin to p65RelA effectively silenced this gene (**Fig. 3A**). Consistent with this, cells with silenced RelA demonstrated reduced transactivation of the NF-kB target gene LIF following TNF-α treatment, compared to vector control (**Fig. 3A**). Notably, despite the significant decrease in p65RelA protein in RelA-silenced H1299 cells, there was no effect on the ability of the P72 variant of p53 to transactivate CSF1 (**Fig. 3B**). To confirm this result, we next sought to pharmacologically inhibit NF-kB and assess the impact on the ability of p53 to transactivate CSF1. Using the IKK inhibitor BAY 11–7082, we were able to show that incubation with this compound effectively inhibited the ability of LIF to be induced following treatment of H1299 cells with TNF-α. In contrast, this inhibitor had no effect on p53-mediated transactivation of CSF1 (**Fig. 3C**). Additionally, we performed chromatin immunoprecipitation for the p53 response element in CSF1 in H1299 tet-inducible p53 cells, stably infected with parental vector or shRelA. As in the transactivation analyses, ChIP experiments revealed there was no effect of silencing p65RelA on the ability of p53 to interact with the CSF1 promoter (**Fig. 3D**). Because the human orthologue of murine caspase-11 has not yet been identified, we were unable to use this gene as a control for these studies. However, the combined data suggest that the P72 variant uses a novel mechanism for increased transactivation of the CSF1 gene. This mechanism involves enhanced ability of P72 to bind to this promoter, and it does not involve increased cooperation of P72 with NF-kB.

### The CSF1 Pathway Enhances p53-mediated Growth Arrest

**Figure 4:**
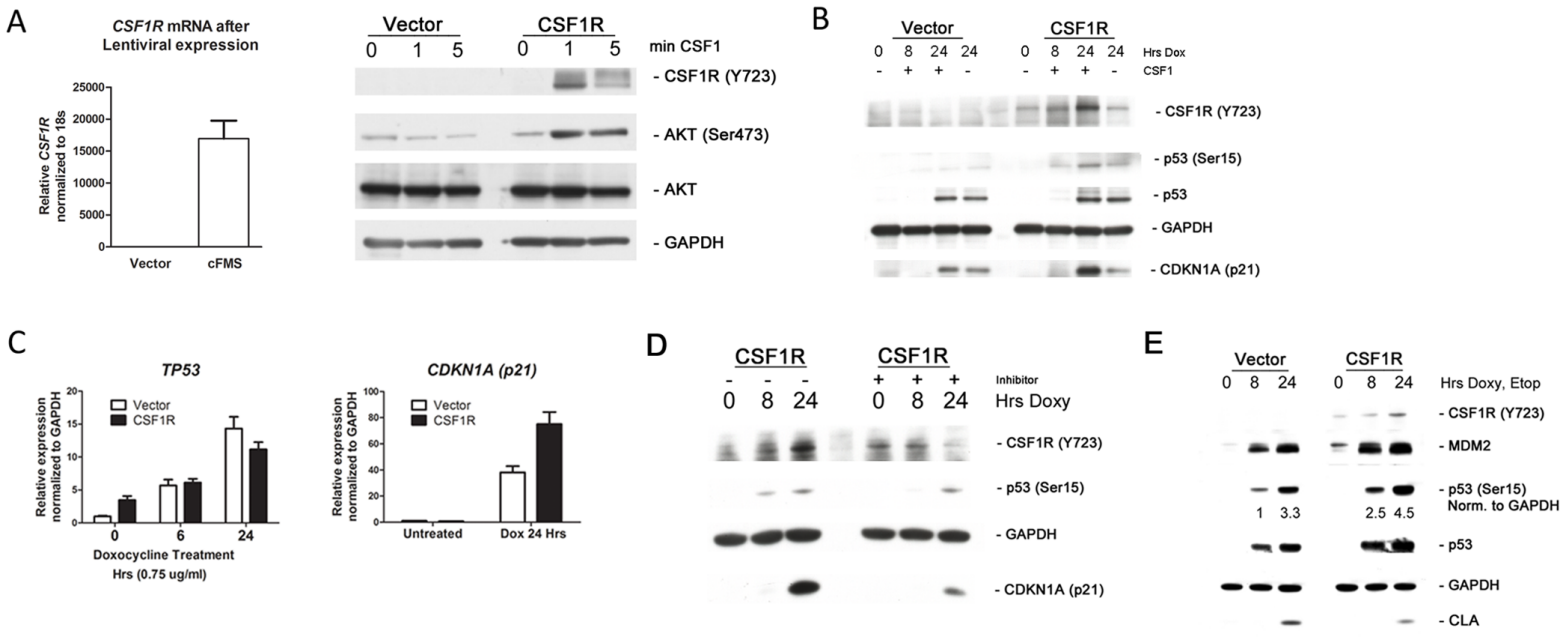
Signaling by CSF1 positively feeds back on the p53 pathway. A. H1299 tetracyline-inducible p53 P72 cells were infected with a lentivirus expressing the CSF1 receptor CSF1R or vector alone (Vector). Left panel: QRT-PCR analysis of stably-infected clones for CSF1R level. Values shown are the average of 2 independent replicates, normalized to control. Error bars mark standard deviation. Right panel: H1299 tetracycline-inducible P72 cells infected with vector (vector) or CSF1R (CSF1R) were treated with 50 ng/mL CSF1 and analyzed for phosphorylated CSF1R protein (phospho-Y723) and AKT (phospho-Ser473) after 0, 1 or 5 minutes. The data depicted are representative of 3 independent experiments. B. H1299 tetracycline-inducible p53 cells stably-infected with vector (Vector) or CSF1R (CSF1R) were serum-starved overnight and then incubated with doxycycline (0.75 ug/ml) alone or in combination with CSF1 (50 ng/ml) for the time points indicated (hours, hrs). Cells were analyzed for activated CSF1R (phospho-Y723), p53 (phospho-Ser15), total p53, MDM2 and GAPDH. The data depicted are representative of 3 independent experiments. C. Left Panel: QRT-PCR of RNA isolated from H1299 tet-inducible P72 cells stably-infected with CSF1R or Vector. Cells were untreated (0 hr) or treated with Doxycycline for the indicated time points. RNA was analyzed for the levels of p53; values shown are the average of 3 independent replicates, normalized to control. Error bars mark standard deviation. Right Panel: QRT-PCR of RNA isolated from H1299 tet-inducible p53 cells (P72 variant), infected with CSF1R or vector. Cells were untreated (0 hr) or treated with doxycycline (0.75 ug/ml) for 24 hours. RNA was analyzed for the levels of p21 (CDKN1A); values shown are the average of 3 independent replicates, normalized to control. Error bars mark standard deviation. D. H1299 tetracycline-inducible p53 cells stably-infected with CSF1R (CSF1R) were treated with or without CSF1R inhibitor II (10 uM, Calbiochem) in combination with doxycycline (0.75 ug/ml) for the time points indicated (hours, hrs). Cells were analyzed for activated CSF1R (phospho-Y723), p53 (phospho-Ser15), GAPDH and p21 (CDKN1A). The data depicted are representative of 3 independent experiments. E. Western analysis of lysates isolated from H1299 tetracycline-inducible p53 cells stably-infected with vector (vector) or CSF1R (CSF1R). Cells were untreated (0 hr) or were treated with doxycycline (0.75 ug/ml) and Etoposide (Etop, 50 uM) for the time points indicated. Cells were analyzed for activated CSF1R (phospho-Y723), p53 (phospho-Ser15), total p53, MDM2, GAPDH and Cleaved Lamin A (CLA). Relative values of Ser15 p53 phosphorylation, assessed by densitometry and normalized to total GAPDH, are indicated.

We next sought to assess the impact, if any, of CSF1 signaling on p53 function. In particular, as CSF1 was previously described as part of a senescence-associated cytokine signature for p53 [29], we sought to test the hypothesis that increased CSF1 transactivation might underlie part of the enhanced ability for the P72 variant to induce growth arrest and senescence, as opposed to apoptosis. Analysis of our inducible H1299 cells revealed an absence of expression of the CSF1 receptor CSF1R in this lung adenocarcinoma background. Therefore, we created a lentiviral expression vector for CSF1R, and infected H1299 tet-inducible P72 cells with this CSF1R construct or vector alone (pLKO-1); we generated pooled stably-infected cells as well as individual sub-clones, and obtained identical results with both (data not shown). To assess the integrity of this pathway in these cells, we first treated vector-infected or CSF1R-infected clones with recombinant CSF1 (50 ng/mL) and measured cFMS activation (Y723 phosphorylation) as well as downstream AKT activation (S473 phosphorylation) using phospho-specific antibodies for these proteins. These analyses suggested that the CSF1/CSF1R pathway was intact in these cells, as assessed by increased CSF1R and AKT phosphorylation after treatment with CSF1 (**Fig. 4A**). We next used doxycycline to induce p53 in these cells in order to analyze the impact of activation of the CSF1R receptor by exogenous CSF1 on the stabilization, phosphorylation, and transcriptional activity of p53. Interestingly, this analysis revealed that CSF1R signaling appears to feed back on the p53 pathway, leading to increased phosphorylation of p53 on serine 15, increased p53 accumulation, and increased expression of the p53 target protein p21 (CDKN1A, **Fig. 4B**). This occurred even in the absence of exogenous CSF1, indicating that the CSF1 induced by p53 was able to signal through this pathway (**Fig. 4B**, compare p53 serine 15 phosphorylation in lanes 4 and 8). The increased expression of p21 was, at least in part, at the transcriptional level, as there was a 2-fold increase in RNA for this gene in the presence of doxycycline in CSF1R cells compared to vector (**Fig. 4C**).

**Figure 5:**
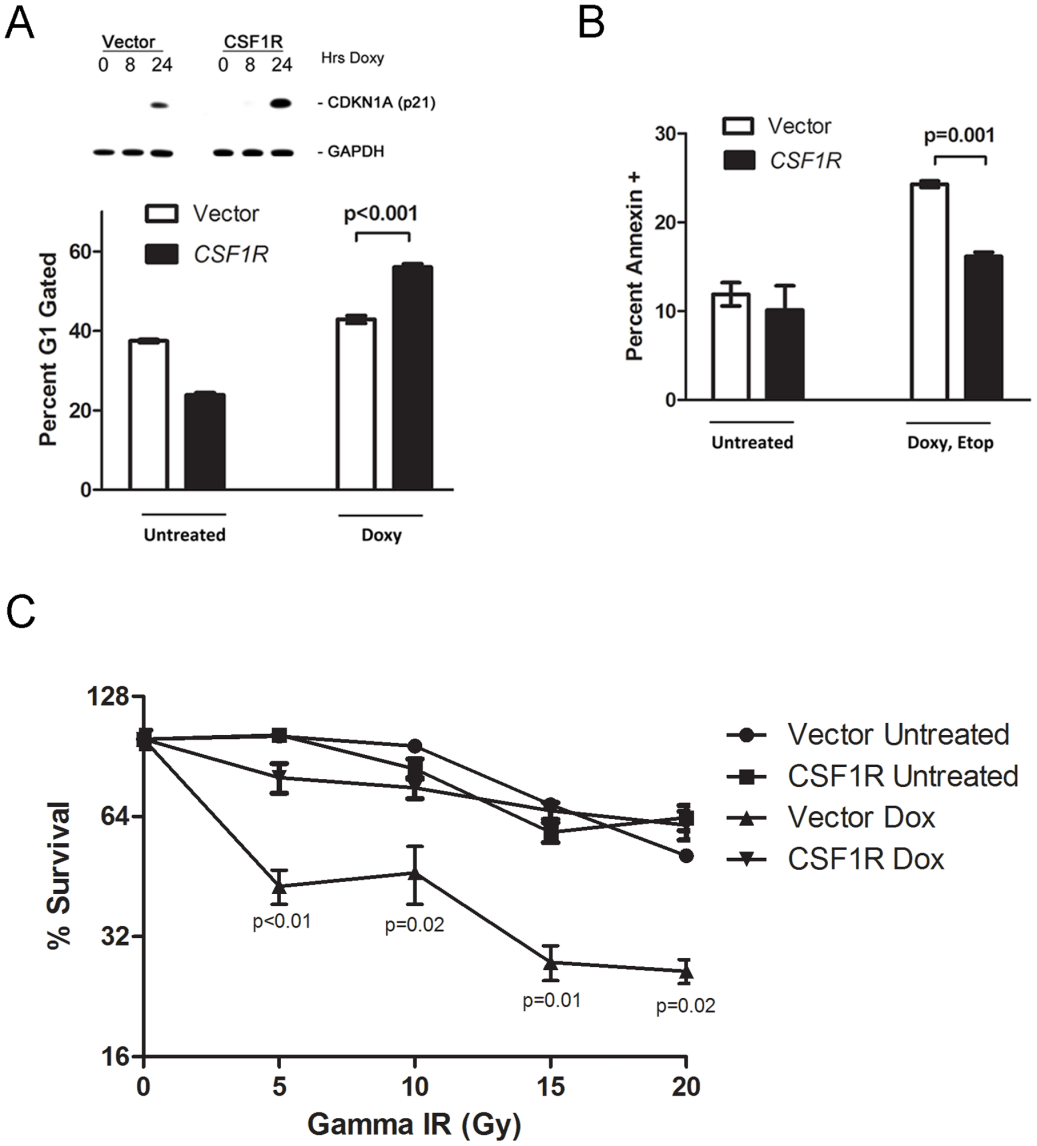
CSF1R signaling promotes cell survival, cell cycle arrest. A. Top panel: Western analysis of lysates from CSF1R overexpressing (CSF1R) or Vector alone (Vector) tet-inducible p53 H1299 cells, untreated (0 hr) or treated with doxycycline (0.75 ug/ml) for the time points indicated (hours, hrs). Cells were analyzed for the levels of p21 (CDKN1A) and GAPDH control. Bottom panel: Flow cytometric analysis of propidium iodide-stained cells from H1299 tetracycline inducible p53 cells stably infected with CSF1R or Vector, untreated or treated with doxycycline (doxy, 0.75 ug/ml) for 24 hours. The percent of cells with 2n DNA content (G1) are shown. Values shown are the average of 3 independent experiments. Error bars mark standard error. B. Flow cytometric analysis of Annexin V-positive cells in H1299 tetracycline inducible p53 cells stably infected with CSF1R or Vector, untreated or treated with doxycycline (doxy, 0.75 ug/ml) and etoposide (50 uM) for 24 hours. Values shown are the average of 3 independent experiments; the p value compares the level of Annexin V positive cells in vector versus CSF1R cells following treatment with doxycycline and etoposide. Error bars mark standard error. C. Colony forming assays were performed with H1299 tet-inducible p53 cells expressing CSF1R (CSF1R) or Vector alone (Vector). Cells were untreated or treated with doxycycline (0.75 ug/ml) 24 hours prior to irradiation with 0, 5, 10, 15 or 20 Gray (Gy) of gamma-irradiation (Gamma-IR). Doxycycline treatments were continued for 8 days; cells were fed every three days. Colonies were then stained with methylene blue and those colonies with greater than 30 cells were counted. Values shown are the average of 3 independent experiments. Error bars mark standard error.

We next sought to confirm that the increased phosphorylation and stabilization of p53, as well as the increased transactivation of p21, was a direct result of increased signaling by the CSF1/CSF1R pathway to p53. Toward this end, we conducted experiments in H1299 tet-on p53 cells expressing the CSF1R, in the absence or presence of the CSF1R inhibitor II (Calbiochem). Notably, in the presence of the CSF1R inhibitor II, the phosphorylation of p53 at serine 15 is reduced, as is the expression of the p53 target gene p21 (**Fig. 4D**). These data confirm that the CSF1/CSF1R pathway signals to induce p53 phosphorylation. We next sought to test the hypothesis that the increased p53 activation in the presence of CSF1/CSF1R signaling might affect the cell fate (cell cycle arrest versus apoptosis) determined by p53. Initially, we tested the level of apoptosis in vector compared to CSF1R cells, and performed these experiments in the presence of doxycycline plus etoposide, in order to increase the number of cells undergoing apoptosis. These experiments revealed that CSF1/CSF1R signaling reduces p53-mediated apoptosis, as evident by decreased cleaved lamin A in the presence of an active CSF1/CSF1R pathway (**Fig. 4E**). To further confirm the impact of CSF1/CSF1R signaling on the p53 growth arrest/cell death decision, we performed flow cytometry of p53-induced cells in vector versus CSF1R-infected cells, and analyzed growth arrest (propidium iodide staining) and apoptosis (Annexin V staining). These studies consistently revealed increased levels of p21, and increased G1 arrest, in CSF1R-expressing cells compared to vector (**Fig. 5A**, p<0.001), along with decreased apoptosis of cells in the presence of etoposide (**Fig. 5B**, p = 0.001). These combined data indicate that the CSF1/CSF1R pathway feeds forward to p53 to enhance p53 signaling and growth arrest, and to inhibit p53-mediated apoptosis.

### The CSF1 Pathway Contributes to Radio-resistance through p53

Expression of the CSF1R is associated with increased radio-resistance of breast cancer cell lines, and high levels of this receptor are associated with local breast cancer recurrence after radiation therapy in women [30], [31], [32], [33]. To test the hypothesis that CSF1/CSF1R signaling through p53 may underlie the increased radio-resistance associated with this pathway in breast cancer cells, we next assessed the radiation sensitivity of our Vector-infected and CSF1R-infected cells, in the presence and absence of doxycycline to induce p53. Colony-forming assays of Vector and CSF1R cells after treatment with increasing doses of gamma radiation revealed that the CSF1R cells consistently showed increased survival after radiation, compared to vector alone; importantly, this effect was entirely dependent on functional p53, because there was no survival benefit in the CSF1R cells in the absence of p53 (**Fig. 5C**). These findings were consistent in multiple independent experiments and several different clones of cells, regardless of whether p53 was induced for the duration of the experiment (eight days), or was restored only transiently with doxycycline (48 hours, **Fig. S3**). The combined data suggest that the ability of CSF1R to upregulate p53, and to enhance p53-mediated cell cycle arrest, may explain the radio-protective effect of this pathway in certain epithelial cancers.

## Discussion

Despite the fact that this polymorphism clearly influences p53′s growth arrest and apoptotic function, the contribution of the codon 72 polymorphism of p53 to cancer risk has been equivocal. In particular, there are as many reports indicating that this polymorphism significantly influences cancer risk, as there are those that indicate there is no impact of this polymorphism on cancer risk (for review see [20]). Confounding factors in these analyses include genetic heterogeneity in human populations, a lack of attention to the latitudinal bias of this polymorphism in control populations [34], and an inability to analyze sufficient numbers of individuals homozygous for the rarer P72 variant. With these limitations in mind, we and our colleagues recently created a mouse model for the codon 72 polymorphism of p53. This model involves a knock-in of human p53 exons 4–9 into the mouse locus [15], [35]. To date, all of the available evidence indicates that this mouse model perfectly recapitulates the findings regarding the function of p53, and codon 72 polymorphic variants, in humans [15], [36], [37], [38]. In this report, we have used this mouse model in the reverse manner: we have uncovered a difference in P72 and R72 function in the mouse thymus (transactivation of CSF1) and found evidence that this difference holds true in human cell lines as well. These data further validate the Hupki mouse model as a discovery tool for the analysis of the impact of genetic alterations on p53 pathway function.

To date, there are few pathways known to influence cell fate (growth arrest versus apoptosis) in response to p53. While there are several cytokine signaling pathways implicated in this decision, until now all of them have been discovered only in hematopoietic cells. For example, M1 myeloid leukemia cells with inducible p53 undergo apoptosis upon p53 induction, and this can be inhibited with the cytokine IL-6; in response to IL-6, p53-mediated growth arrest ensues instead [10]. Similarly, irradiation of bone-marrow derived Baf-3 cells leads to p53-dependent apoptosis, but in the presence of IL-3 these cells instead undergo p53-mediated growth arrest [9]. To our knowledge, CSF1/CSF1R is the first cytokine pathway found to influence the growth arrest versus cell death decision by p53. However, it is almost certain that other growth factor signaling pathways, in other cell types, will be found which also regulate cell fate in response to p53.

Macrophage colony-stimulating factor, or CSF1, has been traditionally viewed as a growth and differentiation factor for monocytes, macrophages and osteoclasts. Both CSF1 and its receptor CSF1R also play roles in trophoblast survival and reproductive development (for review see [33]). Additionally, in some cancers, such as breast, ovarian and endometrial cancer, these proteins are overexpressed, and their expression can be a marker for poor prognosis [30], [32], [39], [40]. In human breast cancer, the levels of CSF1R correlate with local relapse after radiation therapy, but do not correlate with metastasis; these data were the first to suggest that high levels of CSF1R might promote radio-resistance [39]. Consistent with this premise, overexpression of a putative dominant-negative mutant form of CSF1R (Y721F) in mammary epithelial cells increased their sensitivity to radiation; these data indicated that the CSF1/CSF1R pathway could promote radio-resistance [31]. However, the mechanism whereby the CSF1/CSF1R pathway contributes to radiation sensitivity was never elucidated. Here we identify the CSF1/CSF1R pathway as one that functions in a feed forward loop to activate p53, by increasing p53 serine 15 phosphorylation as well as p53 stabilization, along with enhancing p53′s ability to transactivate the cyclin-dependent kinase inhibitor p21 (CDKN1A). Further, we show that signaling from this pathway decreases p53-mediated apoptosis, and concomitantly enhances p53-mediated growth arrest. These data provide a potential explanation for the findings of poor prognosis and relapse after radiation therapy for breast cancers with high levels of CSF1R. These data also suggest that CSF1R inhibitors might be used in order to increase the efficacy of radiation therapy in breast cancers with wt p53 and high levels of CSF1R, particularly in tumors that are homozygous for P72. As the P72 variant is more common in individuals of African descent, these data may have relevance for understanding the poorer prognosis for breast cancer observed in this ethnic group.

Recently, it was reported that in neuroblastoma, another tumor type that rarely contains mutations in p53, the homozygous P72/P72 genotype was associated with decreased apoptosis, along with increased senescence, and poorer prognosis after therapy [41]. Similarly, radiation of normal human fibroblasts that are homozygous for P72 and R72 indicated that P72 fibroblasts exhibited significantly increased senescent cells, compared to R72 [16]. Our data suggest that a re-analysis of the p53 polymorphism status in tumors that rarely mutate p53 should be made, in order to determine the potential impact of the codon 72 polymorphism on radiation response, and to assess the possibility that doses of radiation should be tailored to this genotype.

## Materials and Methods

### Ethics Statement; Animal Studies

This study was carried out in strict accordance with the recommendations in the Guide for the Care and Use of Laboratory Animals of the National Institutes of Health. The protocol was approved by the Institutional Animal Care and Use Committee at the Fox Chase Cancer Center (Permit number 03–11, Animal Welfare assurance number A3285-01). All efforts were made to minimize suffering. P72 and R72 Hupki mice used for thymocyte analysis were generated as previously described [15]. These mice were created in C57BL/6–129 background and backcrossed to C57BL/6 for mice for 7 generations. P72 and R72 mice were then intercrossed for 3 generations, and heterozygotes were mated to yield PP and RR sibling littermates. A cesium-137 gamma radiation source (Fox Chase Cancer Center Irradiation Facility) was used in mouse irradiation experiments; four hours after irradiation, thymuses of mice were collected and thymocytes were extracted. RNA was then harvested from thymocytes and QRT-PCR was performed as detailed below.

### Cell Culture, Drug Treatments, Western Analysis, ELISA

Unless otherwise noted, all cell lines were obtained from the American Type Culture Collection, and cultured cells and frozen vials were used within six months of purchase. Hct116+/+ and −/− cells were generously provided by Bert Vogelstein (Johns Hopkins School of Medicine) [42]. Hct116 p53+/+ and −/− cells, as well as Hep3B and HepG2 cell lines, were cultured in 10% fetal bovine serum (FBS), 1% Pen/Strep in Dulbecco’s Modification of Eagle’s Medium (DMEM, Cellgro, 10-027) with 4.5 g/L glucose and L-glutamine without sodium pyruvate. Tet-inducible p53 P72 and R72 cell lines were provided by Steven McMahon (Thomas Jefferson University) [6], [15] and cultured in Tet-system approved 10% FBS (Clontech, 631106) and 1% Pen/Strep in DMEM. A concentration of 0.75 ug/ml doxycycline was used to induce p53. Adriamycin (Calbiochem, 324380) was administered at a final concentration of 2 uM. Etoposide (Sigma, E1383) was used at a concentration of 50 uM for all experiments. BAY-11-7082 (Cayman Chemicals, 1001026) at a final concentration of 2.5 uM was used. CSF1R inhibitor II (Calbiochem) was used at a final concentration of 10 uM. Human macrophage colony stimulating factor (CSF1, Cell Signaling, 8929LC) was used at the indicated concentrations on cells that had been serum starved for one hour prior to CSF1 addition.

For Western analysis, membranes were blocked, and probed for 1∶5,000 p53 Ab6 (Calbiochem, OP43), 1∶500 p53 ser 15 (Cell Signaling, 9284), 1∶10,000 GAPDH 14C10 (Cell Signaling), 1∶100 p21 Ab6 (Calbiochem, OP79), 1∶200 MDM2 Ab1 and Ab2 (Calbiochem, OP46, OP115), 1∶200 NF-kB p65 (Santa Cruz, C-20), 1∶1000 P-M-CSF Y723 (Cell Signaling, 49C10), 1∶1,000 cleaved lamin A (Cell Signaling, 2035), 1∶1,000 AKT (Cell Signaling, 9272), and 1∶500 P-AKT ser 473 (Cell Signaling, 9271S). Secondary antibodies conjugated to Horseradish peroxidase were used at a dilution of 1∶10,000 (Jackson Immunochemicals). ECL (Amersham, RPN2232) then applied to blots. The Human Macrophage Colony Stimulating Factor (hM-CSF) Ultrasensitive Assay was purchased from Meso Scale Discovery (K151IQC-1) and the assay was performed in accordance with manufactures instructions. P72 and R72 H1299 cells were treated as indicated, collected and lysed; 750 ug of cell lysate was loaded for each sample. The plate was analyzed using the Sector Imager 2400 (Fox Chase Cancer Center).

### RNA Isolation, QPCR

In all cell culture experiments, cells were collected and lysed using Qiashredder columns (Qiagen, 79656). RNA was harvested from lysates using an RNeasy Kit (Qiagen, 74104) including on-column DNase digestion. Equal amounts of RNA from each experiment were then used to create cDNA with the High Capacity cDNA Reverse Transcription Kit (Applied Biosciences, 4368814). Quantitative reverse transcription PCR (qPCR) was performed using Brilliant III Ultra Fast SYBR Green QPCR Mix (Agilent Technologies, 600882) on the Stratagene Mx3005P device (Agilent Technologies). Primers used in qPCR analysis for Hupki studies: MDM2-For 5′AGCGCAAAACGACACTTACA3′, MDM2-Rev 5′ACACAATGTGCTGCTGCTTC3′, CSF1-For 5′ATGGACACCTGAAGGTCCTG3′, CSF1-Rev 5′GTTAGCATTGGGGGTGTTGT3′, Cyclophilin A-For 5′GGGTTCCTCCTTTCACAGAA3′, Cyclophilin A-Rev 5′GATGCCAGGACCTGTATGCT3′. Primers used in qPCR analysis for human cell-line studies: GAPDH-For 5′GAGTCAACGGATTTGGTCGT3′, GAPDH-Rev 5′GACAAGCTTCCCGTTCTCAG3′, CSF1-For 5′ GGAGACCTCGTGCCAAATTA 3′, CSF1-Rev 5′ TATCTCTGAAGCGCATGGTG3′, Puma-For 5′GTCCCCTGCCAGATTTGTC3′, Puma-Rev 5′AGAGGCCGGAGGACACTG3′, Actin-For 5′TTCCTTCCTGGGCATGGAGT3′, Actin-Rev 5′CAGACAGCACTGTATTGGCATA3′, 18S-For 5′GTAACCCGTTGAACCCCATT3′, 18S-Rev 5′CCATCCAATCGGTAGTAGCG3′, RelA-For 5′CCACGAGCTTGTAGGAAAGG3′, RelA-Rev 5′CTGATAGCCTGCTCCAGGTC3′, Lif-For 5′TGTTGGTTCTGCACTGGAA3′, Lif-Rev 5′CCCCTGGGCTGTGTAATAGA3′, CSF1R-For 5′GAGTTCTGCTGCTCCTGCTG3′, CSF1R-Rev 5′CCACACATCGCAAGGTCAC3′, p53-For 5′GTGGAAGGAAATTTGCGTGT3′, p53-Rev 5′CCAGTGTGATGATGGTGAGG3′.

### Lentiviral Transfections and Infections

The ViralPower Lentiviral Expression System and 293FT cells were obtained from Invitrogen and manufacturers instructions were followed for all infections. Lentiviral-encoded shRNA′s specific for RelA in the vector pLKO.1 were purchased from Open Biosystems (RHS3979-9582374). After infection of both P72 and R72 tet-on p53 H1299 cells, RelA-silenced cells were selected for using puromycin (5 ug/ml) for 2 weeks and pooled cell lines were evaluated for RelA expression using western analysis. The CSF1R cDNA clone in vector pReceiver LV-105 was obtained from Capital Biosciences Inc (DHC-4500). After infection of P72 tet-on p53 H1299 cells with CSF1R construct or vector control, cells were selected for using puromycin (5 ug/ml) for 2 weeks.

### Promoter Analysis, Chip-Seq Data Analysis, Chromatin Immunoprecipitation

To identify putative p53 binding sites in genomic sequence, we used a position weight matrix method [43]. The p53 PWM model was built on a curated list of 103 published, experimentally validated p53RE sequences from the literature (NCBI PUBMED database) by converting nucleotide frequency values to position weight matrix score as described [44]. The potential p53-response elements in a genomic sequence were detected by sliding a window along the input sequence, and considering the spacer in p53 binding sites. p53 ChIP-seq data for U2OS cells was from reference [28]. Information on p53 ChIP-Seq from doxorubicin-treated lymphoid cell lines is provided in [27] and reads are in the GEO database. Sequence reads generated from an Illumina Genome Analyzer II were aligned against human reference sequence (GRCh37p5, or hg19, June 2011) using the Burrows-Wheeler Alignment (BWA) Tool. The uniquely mapped short reads were used to identify regions of the genome with significant enrichment in p53-associated DNA sequences. The peak detection was performed by QuEST 2.4 software [45] using the ‘Transcription factor binding site’ setting (bandwidth of 30 bp, region size of 300 bp) and the ‘stringent peak calling’ parameters (corresponding to 50-fold ChIP to input enrichment for seeding the regions and 3-fold ChIP enrichment for extending the regions). The GEO submission numbers for these data are: GSE46991 (p53_DXR_ChIPSeq LCL) and GSE21939 (p53_Eto_ChIPSeq and p53_actD_ChIPSeq, U2OS). Chromatin immunoprecipitation assays were performed as previously described [15]. Antibodies used include: p53 fl393R (Santa Cruz, sc-6243), p53 Ab6 (Calbiochem, OP43), mouse control IgG2a – ChIP grade (Abcam, ab18413). Primers used for evaluating ChIP eluates via qPCR: CSF1-For 5GGAAAGTTCAGGGGGTCAAC3′, CSF1-Rev 5ACCCAAGTCCTCCGTAGCTC3′; MDM2-For 5GGTTGACTCAGCTTTTCCTCTTG3′, MDM2-Rev 5GGCTATTTAAACCATGCATTTTCG3′; p21-For 5AGCAGGCTGTGGCTCTGATT3′, p21-Rev 5CAAAATAGCCACCAGCCTCTTCT3′. Negative control primers IGX1A were purchased from Qiagen (GPH100001C(-)01A).

### Cell Cycle Analysis, Apoptosis Assays, Colony Forming Assays, Statistical Analysis

Evaluation of cell cycle was performed using propidium iodide staining followed by flow cytometry using the Guava easyCyte HT device (Millipore). Annexin staining was performed using Guava Nexin Reagent (Millipore, 4500-0455). For clonogenic survival assays, tetracycline-inducible p53 H1299 cells expressing vector alone (Vector) or CSF1 receptor (CSF1R) were plated at a density of 10^4^ cells in 60-mm culture dishes and incubated overnight in regular media (10% FBS, 1% P/S in DMEM). The following day, cells were treated with or without doxycycline (0.75 ug/ml). After 24 hours cells were irradiated with 0, 5, 10, 15, or 20 Gray (Gy) γ-irradiation using a Cs-137 source (The Wistar Institute). Immediately after irradiation, cells rinsed with phosphate buffered saline (PBS), trypsinized and plated at 5000 cells per well in a 6 well dish. Doxycyline (0.75 ug/ml) was supplemented for 24 hours before irradiation, and either maintained for 48 hours only, or maintained for the duration of the experiment; media was changed every 3 days. After 8 days, cells were washed 3 times with PBS, and stained using 75% methanol with 0.5% crystal violet. Colonies with greater than 30 cells were counted. For percent survival graphs, data were reported as: mean number of colonies/(plating efficiency of non-irradiated control in each treatment group x number of cells plated). Graphpad software package Prism 5 was used for graphing and statistical analysis. Statistical significance was assessed using the paired two-tailed students *t* tests.

## Supporting Information

Figure S1 **p53 binding site in the CSF1 intron 1.** UCSC Browser view of the CSF1 promoter and intronic regions displaying p53 ChIP-seq tracks (red box) from: Upper track: etoposide treated U2OS cells; middle track: Actinomycin treated U2OS cells; lower track: doxorubicin (0.3 uM) lymphoblastoid cells. The lymphoblastoid cell line LCL displayed a 2.4-fold gene expression change at 18 hrs following treatment with doxorubicin (DXR). Below, the sequence within the peak contains 2 overlapping canonical p53 response elements with the calculated position weight matrix (PWM) values indicated.
(TIF)

Figure S2 **P72 shows enhanced binding to the CSF1 promoter, compared to R72.** Quantitative PCR (Q-PCR) analysis of Chromatin Immunoprecipitation (ChIP) eluates isolated from tetracycline-inducible H1299 cells, P72 or R72, untreated or treated with doxycycline (0.75 ug/ml) for 18 hours. CHIP was performed using antibody to p53 (Ab-6, Calbiochem) or an equivalent amount of normal mouse Immunoglobulin G (IgG). Primers used for Q-PCR analysis flank the predicted p53 response element on the CSF1 promoter, or the known p53 response element in the MDM2 promoter. Values shown are the average of 4 independent experiments each repeated in duplicate, and are presented as the relative DNA bound normalized to input. Error bars mark standard error.
(TIFF)

Figure S3 **CSF1R activation promotes cell survival after transient p53 activation.** Colony forming assays were performed with CSF1R overexpressing (CSF1R) or Vector alone (Vector) tet-inducible p53 H1299 cells. Cells were untreated or treated with doxycycline (0.75 ug/ml) 24 hours prior to 0, 5, 10, 15 or 20 Gray (Gy) of gamma-irradiation (Gamma-IR). Doxycycline treatment was continued for 24 hours after irradiation, and then removed. Eight days after irradiation, colonies were stained with methylene blue and those with greater than 30 cells were counted. Values shown are the average of 3 independent experiments. Error bars mark standard error.
(TIFF)
