# Correction: *CSF1* Is a Novel p53 Target Gene Whose Protein Product Functions in a Feed-Forward Manner to Suppress Apoptosis and Enhance p53-Mediated Growth Arrest

**DOI:** 10.1371/annotation/8deafa94-628f-4b03-9dbc-2ec78f5bf737

**Published:** 2013-09-17

**Authors:** Gregory Azzam, Xuting Wang, Douglas Bell, Maureen E. Murphy

There was an error in the Funding statement. The correct version of the statement is available below.

This work was funded by NIH R01 CA102184 (M.M). This work was also funded in part by the Intramural Research Program of the National Institute of Environmental Health Sciences-National Institutes of Health (projects: Z01ES100475 and Z01ES065079). The funders had no role in study design, data collection and analysis, decision to publish, or preparation of the manuscript. 

