# Supplementary figures and images for: CSF1 Is a Novel p53 Target Gene Whose Protein Product Functions in a Feed-Forward Manner to Suppress Apoptosis and Enhance p53-Mediated Growth Arrest
Source: PLoS One. 2013 Sep 3;8(9):e74297. doi: 10.1371/journal.pone.0074297 (PMC3760869; doi:10.1371/journal.pone.0074297)

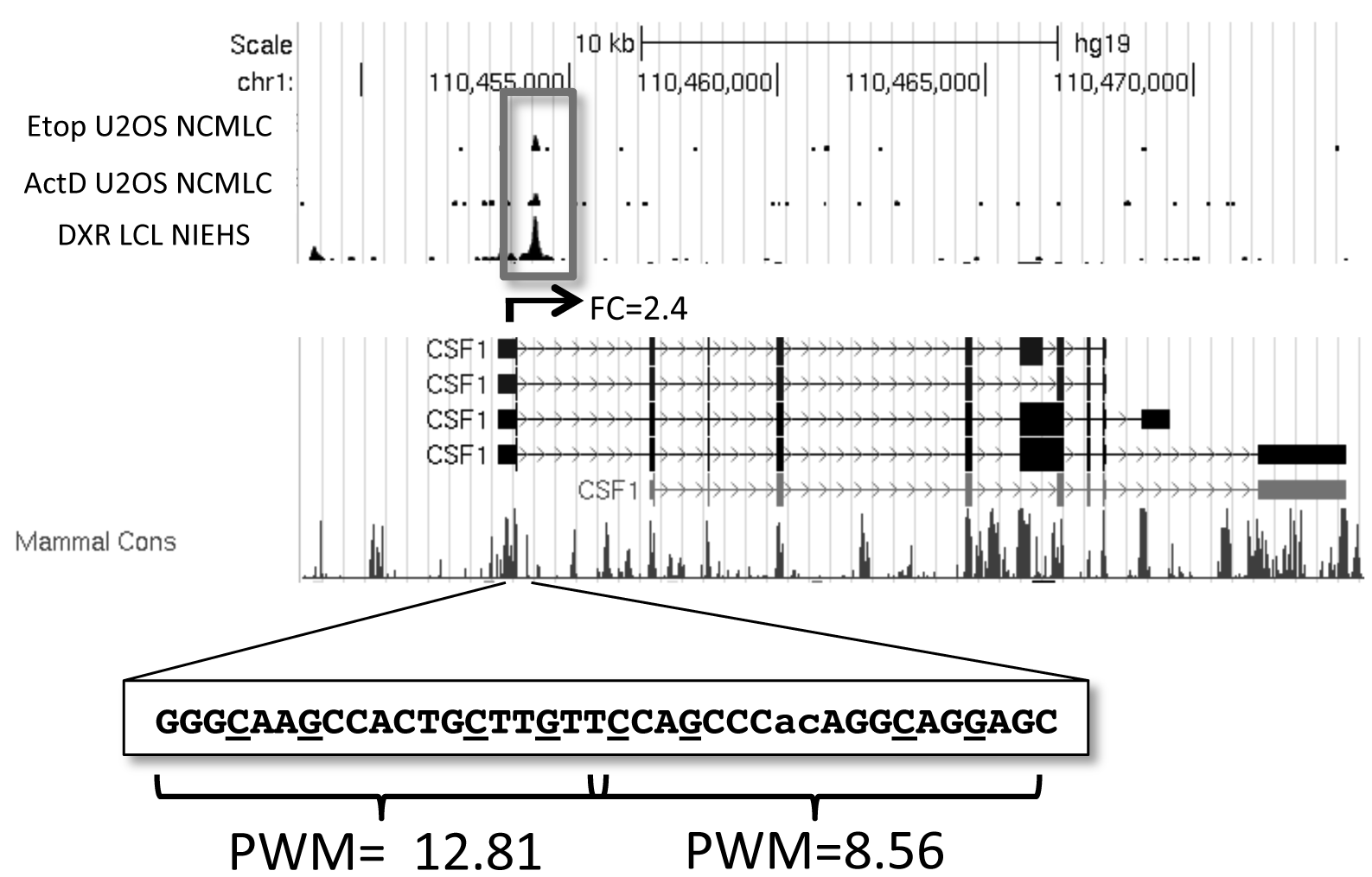

Supplement: Figure S1 — p53 binding site in the CSF1 intron 1. UCSC Browser view of the CSF1 promoter and intronic regions displaying p53 ChIP-seq tracks (red box) from: Upper track: etoposide treated U2OS cells; middle track: Actinomycin treated U2OS cells; lower track: doxorubicin (0.3 uM) lymphoblastoid cells. The lymphoblastoid cell line LCL displayed a 2.4-fold gene expression change at 18 hrs following treatment with doxorubicin (DXR). Below, the sequence within the peak contains 2 overlapping canonical p53 response elements with the calculated position weight matrix (PWM) values indicated. (TIF) [file pone.0074297.s001.tif]

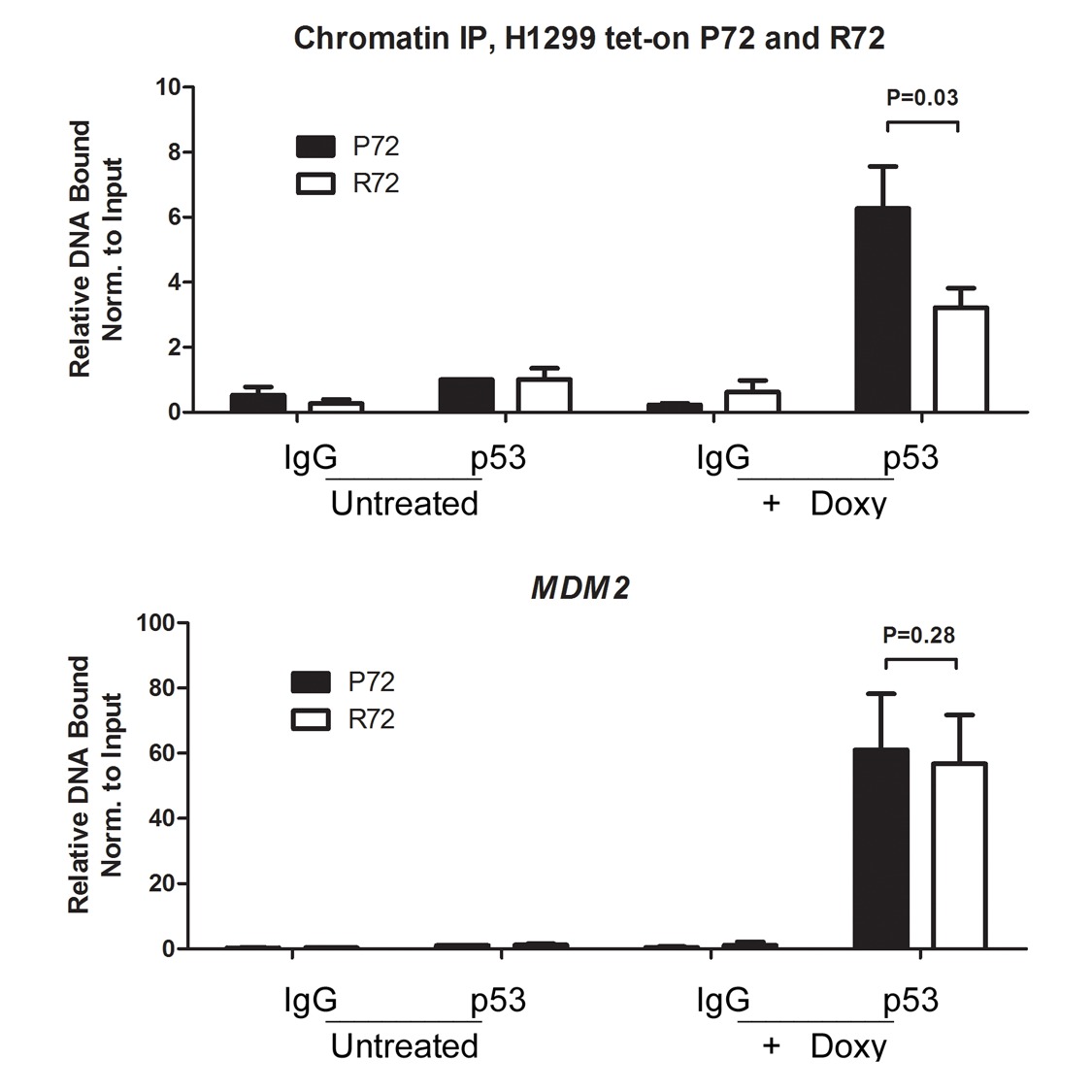

Supplement: Figure S2 — P72 shows enhanced binding to the CSF1 promoter, compared to R72. Quantitative PCR (Q-PCR) analysis of Chromatin Immunoprecipitation (ChIP) eluates isolated from tetracycline-inducible H1299 cells, P72 or R72, untreated or treated with doxycycline (0.75 ug/ml) for 18 hours. CHIP was performed using antibody to p53 (Ab-6, Calbiochem) or an equivalent amount of normal mouse Immunoglobulin G (IgG). Primers used for Q-PCR analysis flank the predicted p53 response element on the CSF1 promoter, or the known p53 response element in the MDM2 promoter. Values shown are the average of 4 independent experiments each repeated in duplicate, and are presented as the relative DNA bound normalized to input. Error bars mark standard error. (TIFF) [file pone.0074297.s002.tif]

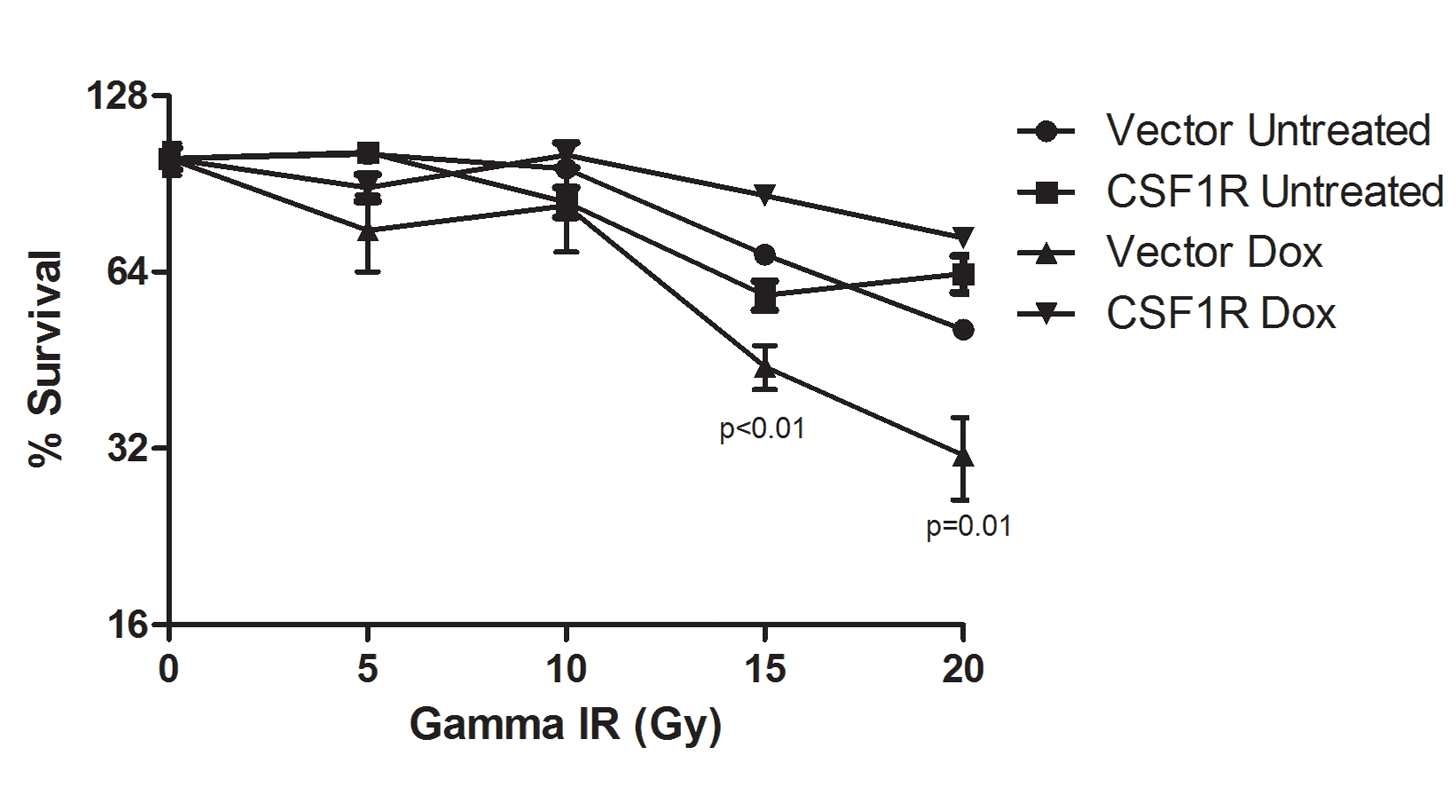

Supplement: Figure S3 — CSF1R activation promotes cell survival after transient p53 activation. Colony forming assays were performed with CSF1R overexpressing (CSF1R) or Vector alone (Vector) tet-inducible p53 H1299 cells. Cells were untreated or treated with doxycycline (0.75 ug/ml) 24 hours prior to 0, 5, 10, 15 or 20 Gray (Gy) of gamma-irradiation (Gamma-IR). Doxycycline treatment was continued for 24 hours after irradiation, and then removed. Eight days after irradiation, colonies were stained with methylene blue and those with greater than 30 cells were counted. Values shown are the average of 3 independent experiments. Error bars mark standard error. (TIFF) [file pone.0074297.s003.tif]
